# Case of Deafness Cured by a Simple Process

**Published:** 1814-05

**Authors:** Charles Morgan


*For the Medical and Physical Journal.*

*Case of Deafness cured by a simple Process; by* Mr. MORGAN.

THE following fact of the cure of Deafness, by a very simple and accidental circumstance, may be worth insertion in your valuable miscellany.

I was consulted by a Barrister in the Temple, for a deafness he had contracted within the last six weeks, and which was occasioned by a common catarrh. With a view to ascertain whether the Eustachian tube was pervious or not, and consequently whether the disease was occasioned by the obstruction of this passage into the ear, I desired him after inspiration to close his mouth and nostrils, and whilst they remained closed, to blow his nose forcibly; instructing him that, if the passage was not obstructed, he would feel a push on the membrane of each ear, from the forcing of the air into the cavity of the tympanum. He did so, and, to his great astonishment, immediately recovered his hearing on the right side; and, by a continuance of the effort a few times, succeeded in clearing the tube on the other side, and is now quite well.

CHARLES MORGAN.

